# Bacterial diversity and prevalence of antibiotic resistance genes in the oral microbiome

**DOI:** 10.1371/journal.pone.0239664

**Published:** 2020-09-29

**Authors:** Viviane de Sousa Moreira Almeida, Jailton Azevedo, Helena Ferreira Leal, Artur Trancoso Lopo de Queiroz, Hermes Pedreira da Silva Filho, Joice Neves Reis

**Affiliations:** 1 School of Pharmacy, Federal University of Bahia, Salvador, Bahia, Brazil; 2 Gonçalo Moniz Research Institute, Oswaldo Cruz Foundation, Salvador, Bahia, Brazil; 3 Health Science Center, Federal University of Recôncavo da Bahia, Santo Antônio de Jesus, Bahia, Brazil; University of the Pacific, UNITED STATES

## Abstract

**Objectives:**

This study aims to describe the oral microbiome diversity and prevalence of ARGs in periodontal health and disease.

**Background:**

The human oral cavity harbors a complex microbial community known as the oral microbiome. These organisms are regularly exposed to selective pressures, such as the usage of antibiotics, which drive evolution and acquisition of antibiotic resistance genes (ARGs). Resistance among oral bacteria jeopardizes not only antibiotic therapy for oral infections, but also extra‐oral infections caused by bacterial translocation.

**Methods:**

We carried out a cross-sectional investigation. Saliva and subgingival plaque samples were collected during a clinical exam. 16S rRNA gene sequencing was performed to assess microbial diversity. Resistance genes were identified through PCR assays.

**Results:**

Of the 110 participants, only 22.7% had healthy periodontium, while the majority was diagnosed with gingivitis (55.4%) and chronic periodontitis (21.8%). The composition of the oral microbiota differed from healthy and diseased samples, being *Streptococcus* spp. and *Rothia* spp. predominant in periodontal disease. Regarding ARGs, 80 (72.7%) samples were positive for at least one of genes screened, *erm* being the most frequent variant (58.2%), followed by *bla*_TEM_ (16.4%), *mec*A (2.7%), *pbp*2b and *aac*(6 ') (1.8%). Neither genes coding resistance to carbapenems nor metronidazole were detected.

**Conclusions:**

Our findings indicate that there are no significant differences in terms of taxonomic enrichment between healthy and diseased oral microbiomes. However, samples retrieved from healthy patients had a more diverse microbial community, whereas diseased samples have lower taxonomic diversity. We have also identified clinically relevant ARGs, providing baseline information to guide antibiotic prescription in dentistry.

## Introduction

The human oral cavity contains a densely-populated microbial ecosystem, termed oral microbiome [1, 2]. Recent deep sequencing analyses estimate the total diversity of this ecosystem in approximately 700 species, including harmless symbionts, commensals, and opportunistic pathogens [3].

The composition of the oral microbiome in healthy adults is generally stable, being *Streptococcus* spp., *Neisseria* spp., *Veillonella* spp., *Actinomyces* spp., the dominant genera [1]. However, microbial diversity may vary due to selective pressures, a few of which are dietary modifications, diseases, and antibiotic exposure [4–6].

While facing selective pressures, microorganisms can express antibiotic resistance genes (ARGs), ensuring their survival and genetic persistence. As a result, the oral cavity becomes an abundant source of ARGs, increasing the risk of resistant bacterial infections [5, 6].

Resistance among oral bacteria limits therapeutic options and affects treatment success rates for oral infections [7–9]. Moreover, it has also extra‐oral implications. Typically, healthy periodontal tissues prevent active microbial invasion. However, periodontal diseases and invasive procedures often lead to tissue damage. Injured epithelia enable the passive transfer of oral bacteria to the bloodstream, whereby they can translocate and reach other body sites [10, 11].

Periodontal diseases (PD), namely gingivitis and periodontitis, are the most prevalent chronic oral infections, affecting millions worldwide [12, 13]. These illnesses are characterized by inflamed gingival tissue owing to dental plaque accumulation. Severe cases are associated with gingival recession, loss of tissue and alveolar bone damage [14].

Current evidence indicates that PD onset stems from shifts in microbial balance (dysbiosis), rather than being caused by a singular pathogenic agent [4, 15–19]. Therefore, to prevent these diseases and their systemic implications, it is necessary to unravel the complex microbial interactions that lead to the transition from health to disease.

In order to provide insights into this dynamic, we carried out a comprehensive evaluation of oral microbiome diversity and the prevalence of ARGs in healthy and diseased subjects.

Many previous studies evaluating the prevalence of ARGs in oral bacteria are based on cultivable isolates solely, not taking into consideration a large number of non-cultivable bacteria in these communities [20, 21]. To address this issue, we used cultivation-independent molecular methods. Furthermore, we looked into demographics, clinical history, and personal habits aiming to identify risk factors for PD and ARGs carriage.

We hope that the evidence presented here broadens our understanding of the etiology of periodontal diseases, as well as supports effective antimicrobial therapy in dentistry.

## Methods

### Study design and population

We set up a cross-sectional study. Subjects were recruited from October 2016 to November 2016 at the Graduate School of Dentistry and the Laboratory of Clinical Analysis from the Federal University of Bahia (UFBA), Brazil.

The population was composed of patients of both sexes, who were 18 years old or older, and who were willing to participate in the investigation. Individuals who had undergone antibiotic therapy in the prior 30 days were excluded. All patients enrolled in this research (*n* = 110) provided written informed consent. All procedures performed in this study met institutional and national ethical standards and were approved by the Human Research Ethics Committee of the Pharmacy School of the Federal University of Bahia (UFBA), Brazil (approval nº 1.756.977).

### Clinical procedures and sample collection

We carried out a comprehensive periodontal evaluation by examining gingival bleeding, calculus accumulation, probing depth, furcation involvement, tooth mobility, mucogingival problems, and gingival recessions.

The Periodontal Screening and Recording (PSR) system was used to determine the periodontal status [22]. Participants were classified into three groups:

- Healthy periodontium (PSR0): no clinical signs of disease; colored area of probe remains completely visible; no calculus or bleeding are detected.
- Gingivitis (PSR1 or PSR2): Bleeding in more than 20% of sites after 30s of gentle probing, colored area of probe remains completely visible in the deepest probing depth in the sextant. Supra- or subgingival calculus are detected.
- Chronic periodontitis (PSR3 or PSR4): Bone loss greater or equal to 3.5mm.

We evaluated mesiobuccal, vestibular, distal-vestibular, mesiolingual, lingual, and distal-lingual sites of each tooth, excluding third molars. All clinical procedures were performed by the same dentist. Exams were executed under artificial light using a plane mouth mirror, and a World Health Organization (WHO) dental probe.

Subsequently, saliva samples were collected using the spitting method. The dentist instructed volunteers to do not eat, drink, smoke, or chew gum for 30 minutes beforehand. The patients were asked to rinse their mouths with bottled water to remove food debris. Then, they were asked to forcefully spit saliva (not sputum or foam) directly into an appropriate sterile container [23, 24].

Afterwards, subgingival dental plaque samples were collected using sterile Gracey curettes (Hu-Friedy). Saliva samples and dental plaque specimens were pooled to create a single sample for each patient. All flasks were properly labeled and stored at −80°C until DNA extraction.

### DNA extraction and detection of Antibiotic Resistance Genes (ARGs)

The DNA extraction was performed using the Maxwell RSC DNA Kit (Promega, Germany) using the Maxwell RSC Blood DNA Kit. Saliva samples (400 μl) were incubated with 30 μl lysozyme and 300 μl lysis buffer for 2 hours at 37°C. The samples were then added to the cartridges and automatically processed according to the manufacturer’s instructions. DNA was eluted in 80 μl of Maxwell elution buffer. Its purity and concentration were measured using a NanoDrop™ spectrophotometer.

Subsequently, real-time Polymerase Chain Reactions (PCR) were carried out using a 16S ribosomal RNA gene probe. This procedure aimed to identify which clinical samples contained bacterial DNA [25]. Positive samples were defined as those with a Threshold Cycling Value (CT) <20.

Then, the following ARGs were screened by PCR: *aac*(6') [26], *bla*_TEM_, *bla*_SHV_, *bla*_OXA-1-like_ [27], *bla*_CTX-M_, *bla*_KPC_ [28], *bla*_IMP_, *bla*_VIM_ [29], *bla*_NDM_ [30], *bla*_OXA-48-like_ [31], *pbp*2b [32], *mec*A [33], *erm* [34], and *nim* [35]. We used primer sequences and protocols described by the cited authors. The positive controls were bacterial DNA extracts from reference strains. All experiments were run with PCR conditions and primers previously reported to the literature and are described on the S1 Table.

### 16S rRNA gene sequencing

A subset of samples was randomly selected using an R studio command (https://stat.ethz.ch/R-manual/R-devel/library/base/html/sample.html) for microbiota characterization using 16S rRNA gene sequencing.

Genomic DNA was used as a template to amplify the V3/V4 variable region of 16S rRNA genes using the primers: 341F (CCTAYGGGRBGCASCAG) and 806R (GGACTACNNGGGTATCTAAT) [36]. PCR reactions were performed using the Phusion® High-Fidelity PCR Master Mix (New England Biolabs), 0.5 μM of each primer, and 100ng of DNA. Amplification was carried out as follows: initial denaturation at 98°C for 30 s; 25 cycles of 98°C for 10 s, 50°C for 10 s and 72°C for 10 s; and a final elongation step at 72°C for 7 min.

Amplicons were purified using the Qiagen Gel Extraction Kit (Qiagen, Germany) according to manufacturer's guidelines. Sequencing libraries were generated using the NEBNext® UltraTM DNA Library Prep kit for Illumina (New England Biolabs), and index codes were added. The library quality was assessed using the Qubit@ 2.0 Fluorometer (Thermo Scientific) and the Agilent Bioanalyzer 2100 system. Lastly, the library was sequenced on the Illumina HiSeq2500 platform, and 250bp paired-end reads were generated.

### Data analysis and taxonomic assignment

The data was entered, managed, and analyzed using Epi Info version 3.5.1 (CDC, Atlanta, GA, USA).

Clinical features were analyzed using non-parametric tests. Non-normal variables were reported as median and interquartile ranges (1qt–3qt). Fisher’s exact or Mann-Whitney U tests were used to compare the differences between the proportions for dichotomous variables. Odds ratio (OR) and 95% confidence intervals (CIs) were calculated as measures of association. Statistical significance was defined as *P*<0.05.

Operational taxonomic unit (OTU) clustering was performed at a 97% identity threshold using the UPARSE pipeline. Representative sequences were classified into organisms using the RDP Classifier based on the Green genes database (version gg-13-5).

For plotting bacterial composition and comparison between the groups, the data were transformed to the compositional relative abundance with the function transform, from the microbiome package in R 3.5.1.

The complexity of microbial communities was measured using Alpha-Diversity and Beta-Diversity methods. Alpha-diversity evaluates the variety and abundance of organisms in a single community/sample. On the other hand, Beta-diversity assesses the differences among multiple microbial communities/samples.

For Alpha analyses, boxplots were generated using the *diversities* function from the *microbiome* package in R 3.5.1., which is available online at https://microbiome.github.io/tutorials/”. Beta-analyses were performed using *ordination* and *plot ordination* functions from the *phyloseq* package in R 3.5.1 [36]. The dissimilarity between every pair of community samples was calculated using a square matrix of distance. Group differences were calculated using the *stats* package in R 3.5.1. T-test and Wilcox were used for comparing two groups, whereas ANOVA and Tukey tests were employed for analyzing three groups or more.

## Results

### General characteristics of the study population

From October 2016 to November 2016, 110 individuals were enrolled in the study. Most of the patients were female (79.0%, 87/110) and to be middle-aged (median age 41.5 years). The characteristics of the participants are shown in Table 1.

**Table 1:** Demographic and clinical features of patients stratified by periodontal health status (*n =* 110).

Characteristics	Healthy periodontium	Periodontal disease	
	*n* (%)	*n* (%)	
	25 (22.7)	85 (77.3)	
		Gingivitis	Chronic periodontitis
		61 (55.5)	24 (21.8)
**Demographic data**			
*Sex*			
Female	22 (88.0)	49 (80.3)	16 (66.7)
Male	3 (12.0)	12 (19.7)	8 (33.3)
*Age*			
Age groups (years), median (1qt-3qt)	38 (20–66)	43 (18–71)	43 (21–60)
18–35	10 (40.0)	19 (31.1)	7 (29.2)
36–50	7 (28.0)	24 (39.3)	11 (45.8)
>50	8 (32.0)	18 (29.5)	6 (25.0)
*Self-reported race*			
Black	18 (72.0)	30 (49.1)	12 (50.0)
Brown	6 (24.0)	26 (42.6)	9 (37.5)
White	1 (4.0)	5 (8.2)	3 (12.5)
*Education*			
High school	14 (56.0)	32 (52.4)	8 (33.0)
College education	1 (4.0)	6 (9.8)	1 (4.2)
Per capita monthly income*			
1–2 minimum wages	23 (92.0)	41 (67.2)	14 (58.3)
3–5 minimum wages	1 (4.0)	11(18.0)	6 (25.0)
6–7 minimum wages	-	3 (4.9)	-
No income	-	6 (9.8)	3 (12.5)
**Underlying diseases**			
Diabetes	3 (12.0)	3 (4.9)	2 (8.3)
Hypertension	3 (12.0)	11 (18.0)	6 (25.0)
Cardiopathy	1 (4.0)	1 (1.6)	6 (25.0)
**Smoking history**			
Smokers	-	3 (4.9)	-
Ex-smokers (less than 10 years)	3 (12.0)	3 (4.9)	4 (16.7)
Non-smokers	22 (88.0)	55 (90.2)	20 (83.3)
**Pregnancy** **	3 (12.0)	4 (6.5)	7 (29.2)
**Clinical information**			
Mean number of teeth (mean ± SD)	22.7 (10.3)	16.3 (8.7)	16.2 (5.4)
Mean number of decayed teeth (mean ± SD)	0.84 (1.7)	1.1 (1.6)	1.7 (2.3)
Use of partial prosthesis	10 (40.0)	15 (24.6)	8 (33.3)
Dental plaque	-	44 (72.1)	20 (83.3)
Bleeding on probing (BOP)	-	30 (49.0)	9 (37.5)
Mean number of sextants with pockets	-	-	1.5 (1.0)

(-) Zero

(*) The Brazilian minimum wage was R$ 880.00, equivalent to US$270.00, in 2016

(**)Of the 87 female patients, 14 were pregnant; (SD) Standard deviation.

Only 22.7% (25/110) of the patients had a healthy periodontium, while the majority was diagnosed with periodontal diseases (77.3%, 85/110). Gingivitis was identified in 55.4% (61/110) subjects and chronic periodontitis in 21.8% (24/110).

Univariate analyses were performed to investigate the possible risk factors associated with periodontal disease. No significant differences were observed regarding sex, age, self-reported race, *per capita* monthly income, underlying diseases, or smoking history.

Regarding indicators of oral health, the number of preserved teeth (mean± SD) was higher in healthy patients (22.7, 10.3). Similarly, the number of decayed teeth (mean± SD) was lower among this group (0.84, 1.7). However, statistical significance was not reached for any of these associations.

Since the presence of dental plaque, bleeding on probing (BOP), and the number of sextants with pockets are clinical parameters for diagnosing periodontal disease, they were not evaluated as risk factors. Therefore, we did not estimate Odds Ratio (OR) in these cases.

### Prevalence of antibiotic resistance genes

Overall, 80 of 110 (72.7%) samples were positive for at least one of the ARGs screened, *erm* being the most frequent variant (58.2%, 64/80), followed by *bla*_TEM_ (16.4%, 18/80), *mec*A (2.7%, 3/80), *pbp*2b and *aac* (6 ') (1.8%, 2/80) (S1 Table). The genetic elements *bla*_SHV_, *bla*_OXA-1-like_, *bla*_*CTX-M*_, *bla*_KPC_, *bla*_IMP_, *bla*_VIM_, *bla*_NDM_, *bla*_OXA-48-like_, and *nim* were not detected. The prevalence of ARGs stratified by periodontal health status is presented in Table 2.

**Table 2:** Prevalence of Antibiotic Resistance Genes (ARGs) stratified by periodontal health status (*n* = 110).

ARGs	Healthy periodontium	Periodontal disease		All patients
	*n* (%)	*n* (%)		*n* (%)
	25 (22.7)	85 (77.3)		110 (100)
		Gingivitis	Chronic periodontitis	
		*n* (%)	*n* (%)	
		61 (55.5)	24 (21.8)	
*erm*	15 (60.0)	35 (57.4)	14 (58.3)	64 (58.2)
*bla*_TEM_	2 (8.0)	13 (21.3)	3 (12.5)	18 (16.4)
*aac(6´)*	1 (4.0)	1 (1.6)	0 (0.0)	2 (1.8)
*pbp2b*	1 (4.0)	1 (1.6)	0 (0.0)	2 (1.8)
*mecA*	0 (0.0)	0 (0.0)	3(12.5)	3 (2.7)

Of the 110 samples recovered, 80 (72.7%) were positive for at least one of the tested genes. In 9 cases two resistance genes were found in the same sample. Overall, 89 ARGs were detect.

The occurrence of more than one ARG within the same sample has been detected in 9 cases. Five samples (4.5%, 5/110) carried the gene *erm* in association with *bla*_TEM_. All samples positive for the gene *mec*A also carried the gene *erm*. One sample carried the *pbp*2b and the *erm* genes together. The proportion of samples positive for ARGs was similar between healthy (18/25, 72.1%) and diseased (62/85, 72.9%) patients.

### Oral microbiome analysis

We analyzed the taxonomic composition and abundance of the oral bacterial community from 22 subjects (20%, 22/110) using barcoded pyrosequencing of the 16S rRNA gene.

The samples were selected based on their resistance patterns. Among them, 4 (18.2%, 4/22) were positive for the *erm* gene, 6 (27.3%, 6/22) for the *bla*_TEM_ gene, 2 (9.1%, 2/22) for *mec*A and *pbp*2 genes, and 10 (45.5%, 10/22) were negative for all ARGs investigated. These categories were termed “ERM group”, “TEM group”, “Others group”, and “SUSC group” respectively.

Of the 22 samples, 4 (18.2%, 4/22) were retrieved from healthy patients, 12 (54.5%, 12/22) from those who diagnosed with gingivitis and 6 (27.3%, 6/22) from those with chronic periodontitis.

The mean number of reads per sample assigned to OTUs was 46982, ranging from 46k to 48k. On average, 620 OTUs were identified and classified into 33 genera or higher taxa.

**Fig 1:**
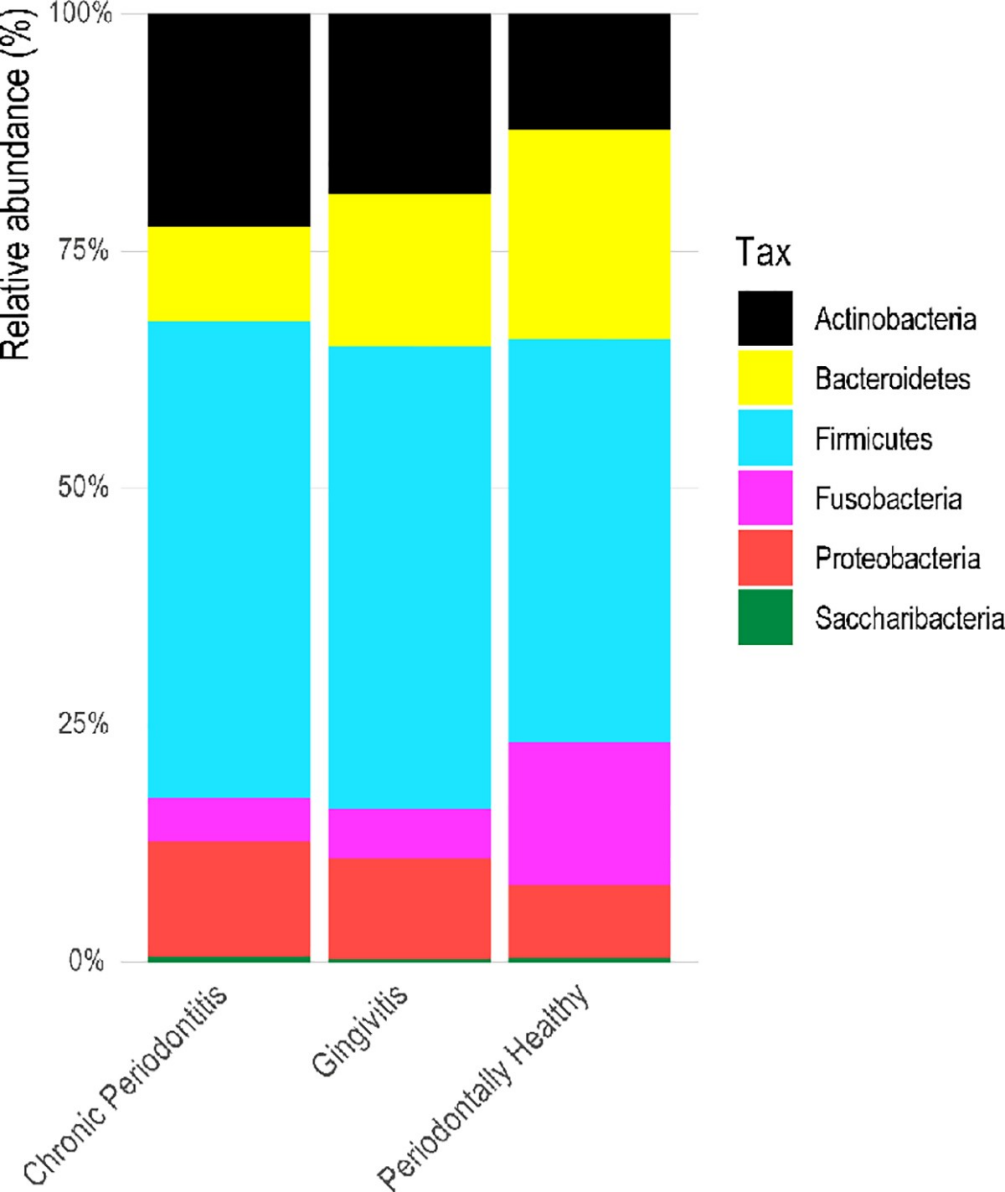
Relative proportion of bacterial phyla in healthy and diseased samples. Relative proportion refers to the percentage of each phyla (indicated by different colors) in a resistance group. Overall, 22 samples were analyzed being 4 (18.2%) retrieved from healthy patients, 12 (54.5%) from those with gingivitis, and 6 (27.3%) from those with chronic periodontitis.

**Fig 2:**
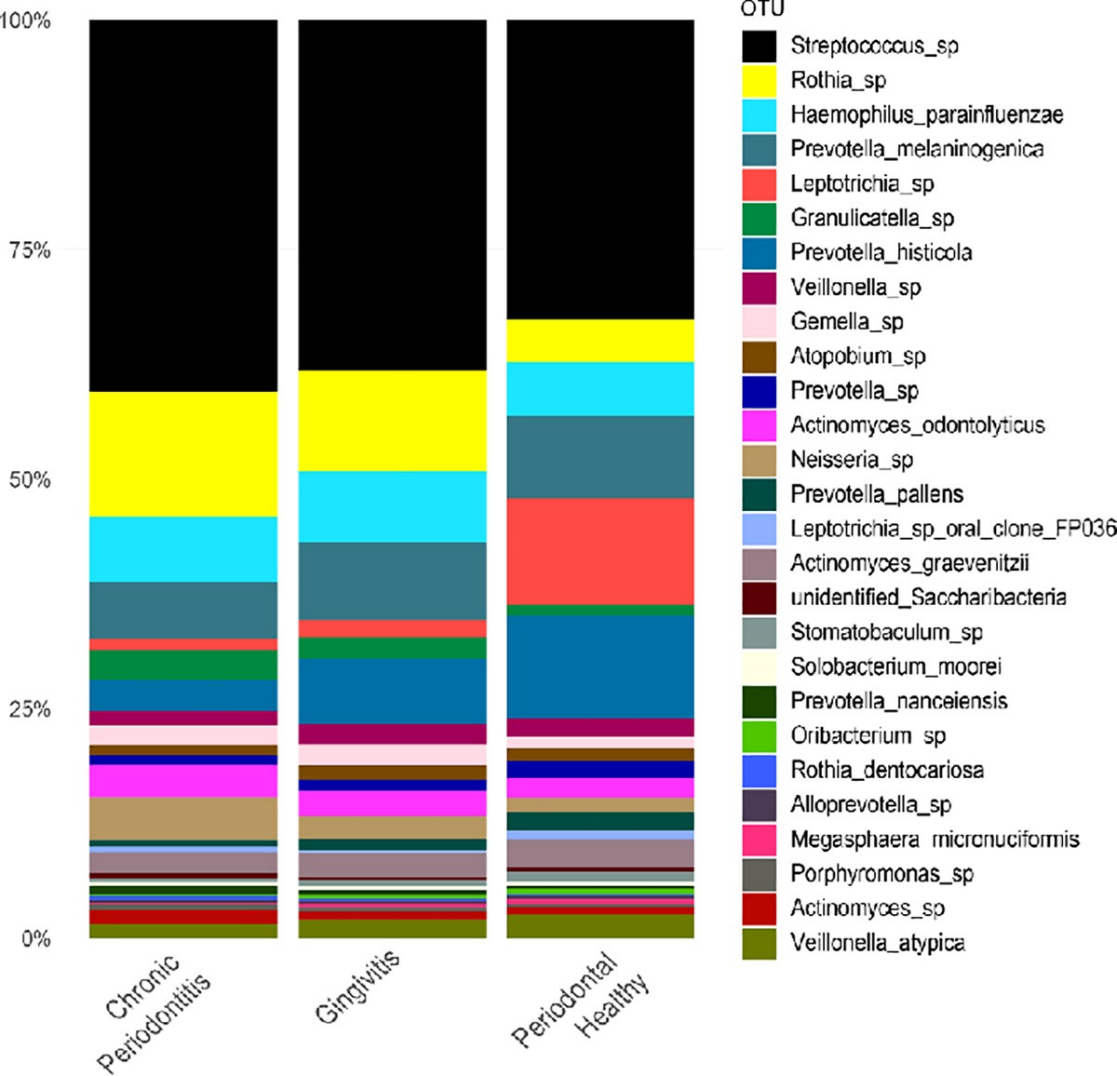
Relative proportion of oral bacteria in healthy and diseased samples. Relative proportion refers to the percentage of each genus/species (indicated by different colors) in a patient group. Overall, 22 samples were analyzed being 4 (18.2%) retrieved from healthy patients, 12 (54.5%) from those with gingivitis, and 6 (27.3%) from those with chronic periodontitis.

Taxonomic profiling of the samples, whether from healthy or diseased patients, reveals a community dominated by the phylum *Firmicutes*. The phyla *Bacteroidetes* and *Fusobacteria* are relatively increased in healthy samples. On the other hand, the phyla *Actinobacteria* and *Proteobacteria* are more abundant in diseased samples (Fig 1). Multiple pairwise comparisons show that the phyla *Actinobacteria* and *Bacteroidetes* were significantly strongly correlated to diseased and healthy samples, respectively (S1 Fig). At the genera level, *Streptococcus* and *Rothia* dominated both healthy and diseased samples (Fig 2).

**Fig 3:**
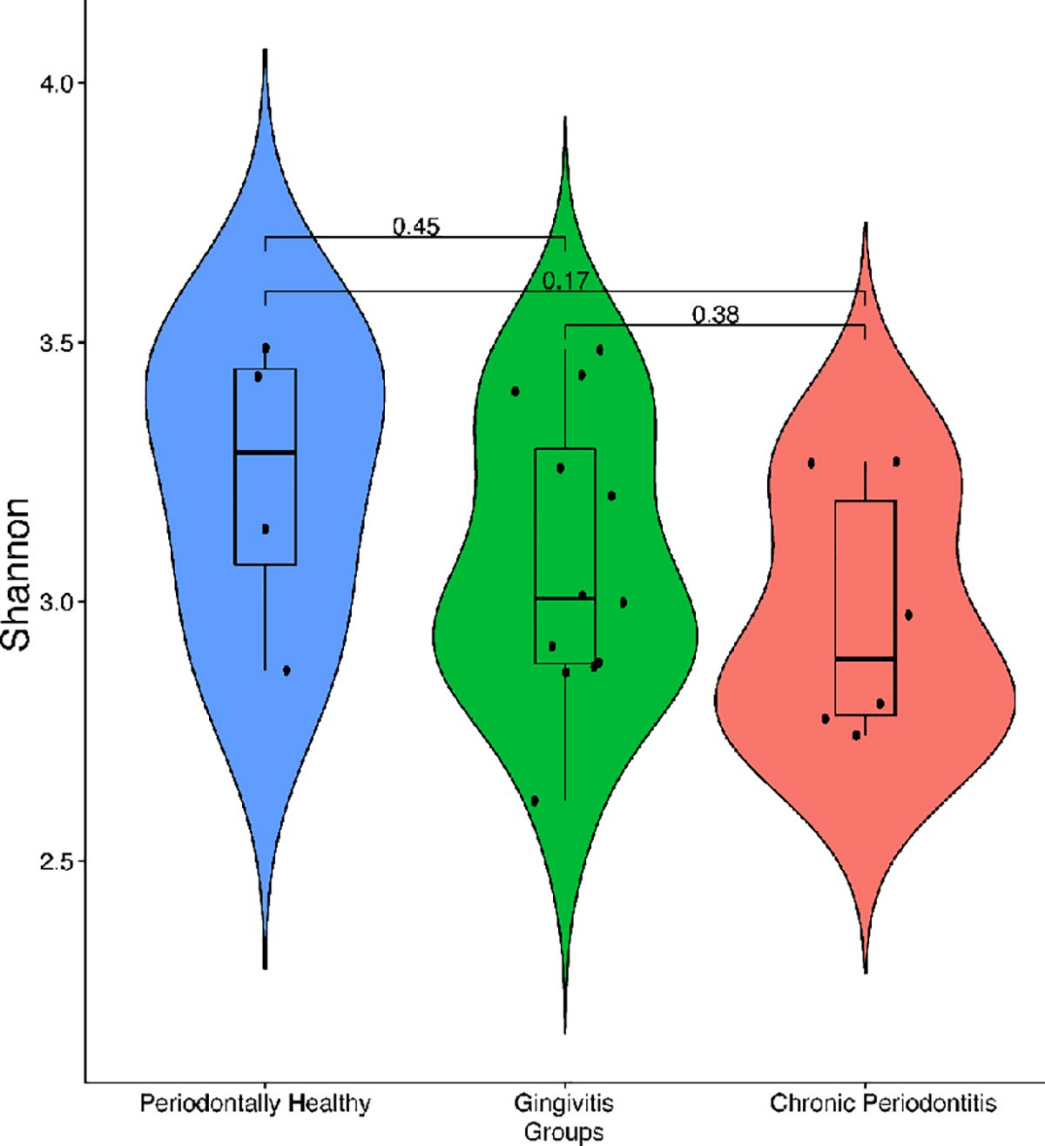
Taxonomic diversity of bacterial communities in healthy and diseased samples. Violin plots show the distribution and density of oral bacteria across three groups of patients. Overall, 22 samples were analyzed being 4 (18.2%) retrieved from healthy patients, 12 (54.5%) from those with gingivitis, and 6 (27.3%) from those with chronic periodontitis. Alpha diversity was measured using the Shannon index (Y-axis). The boxes represent the interquartile range (IQR) between the first and third quartiles (25th and 75th percentiles, respectively). The vertical line inside the box defines the median. Whiskers represent the upper and lower adjacent values. *P*-values of comparisons are displayed above each block analyzed.

However, when comparing the different groups of patients, we observed a shift in the composition of the oral microbiota. Healthy samples harbor a more diverse microbial community, whereas diseased samples have lower taxonomic diversity (Figs 2 and 3).

Even though the genera *Streptococcus* and *Rothia* were identified in healthy samples, they were more abundant in periodontal disease. Meanwhile, *Leptrotrichia* and *Prevotella histolitica* were found at higher levels in healthy patients (Fig 3). Nevertheless, it is important to clarify that none of these associations were statistically significant.

**Fig 4:**
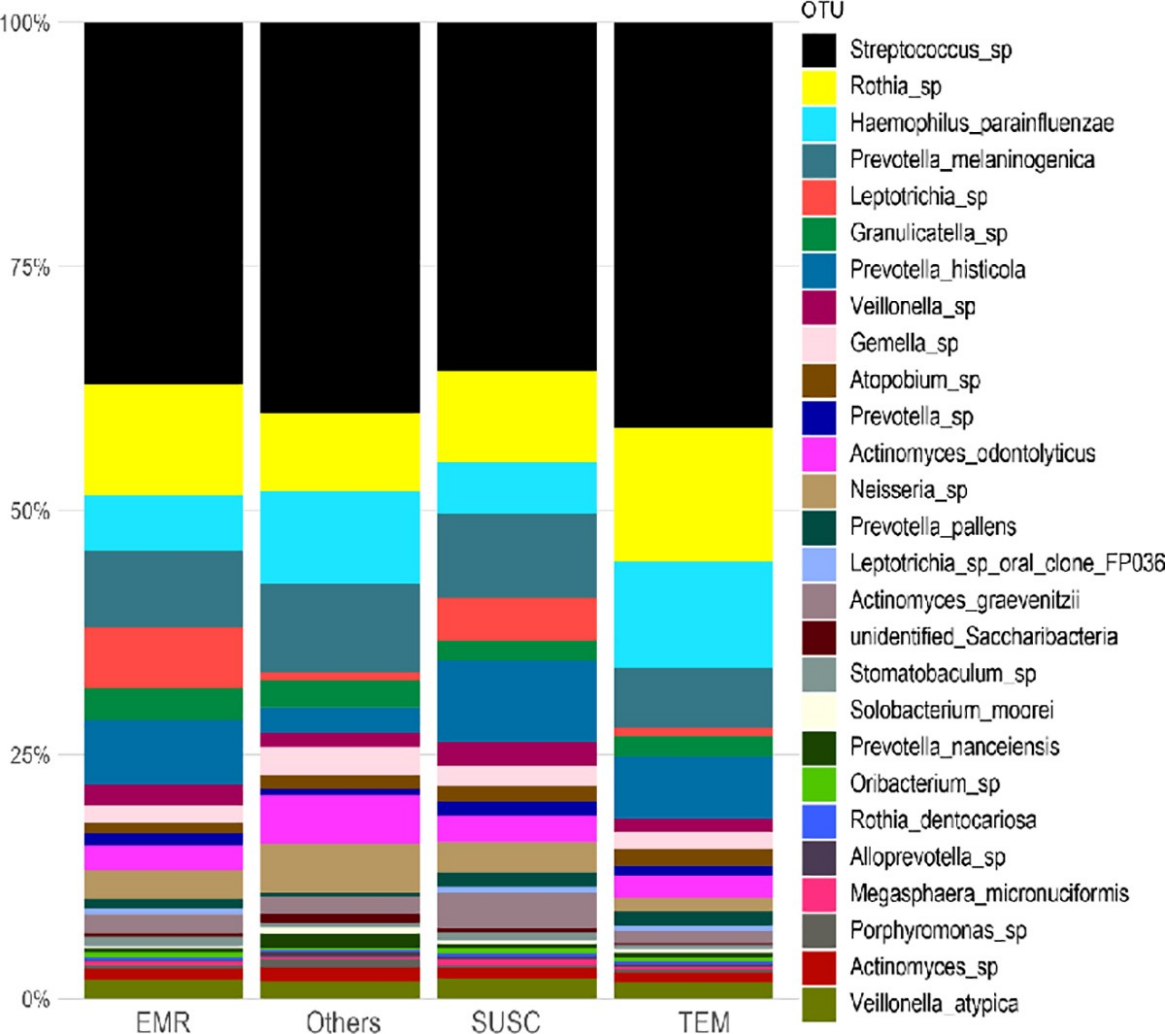
Relative proportion of oral bacteria stratified by resistance groups. Relative proportion refers to the percentage of each genus/species (indicated by different colors) in a resistance group. Overall, 22 samples were analyzed being 4 (18.2%) positive for the *erm* gene (EMR group), 6 (27.3%) for the *bla*_TEM_ gene (TEM group), 2 (9.1%) for *mec*A and *pbp*2 genes (Others group), and 10 (45.5%) negative for all searched genes (SUSC group).

We have also examined associations between resistance patterns and specific microbial community structures. The Fig 4 illustrates the relative proportion of oral bacteria stratified by resistance groups (ERM, TEM, Others, and SUSC).

The genera *Streptococcus*, *Rothia*, *Neisseria* and *Granulicatella* were dominant in all groups, while *Leptotrichia* was enriched in EMR and SUSC groups. At the species level, *Haemophilus parainfluenzae*, *Prevotella melaninogenica*, *Prevotella histolitica*, and *Actinomyces odontolyticus* were abundant in all groups.

**Fig 5:**
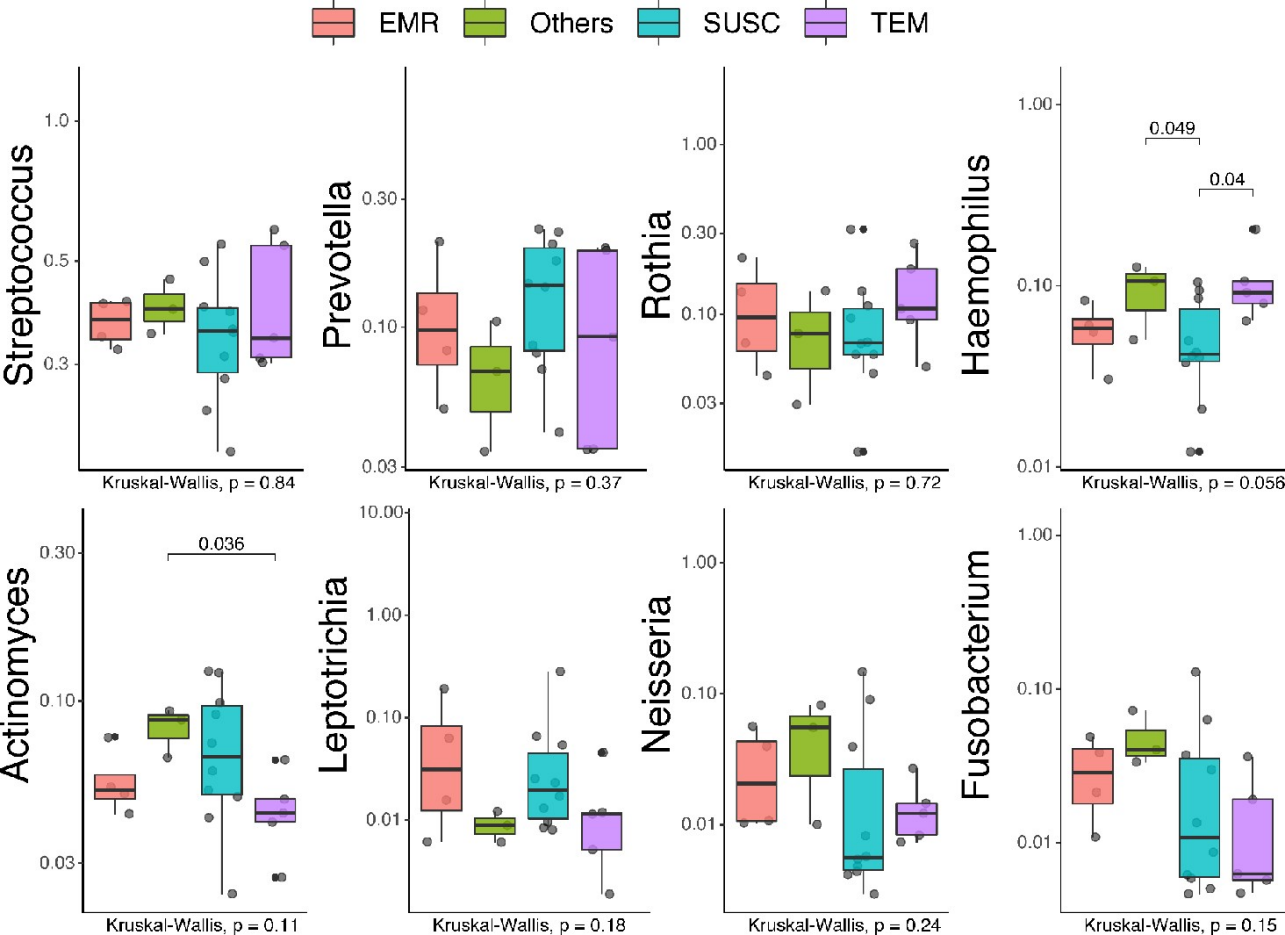
Distribution of bacterial genera/species stratified by resistance groups. Box plots illustrate the abundance of selected genera/species across resistance groups. Overall, 22 samples were analyzed being 4 (18.2%) positive for the *erm* gene (EMR group), 6 (27.3%) for the *bla*_TEM_ gene (TEM group), 2 (9.1%) for *mec*A and *pbp*2 genes (Others group), and 10 (45.5%) negative for all searched genes (SUSC group). The boxes represent the interquartile range (IQR) between the first and third quartiles (25th and 75th percentiles, respectively). The vertical line inside each box defines the median. Whiskers represent the upper and lower adjacent values. Gray circles are outliers. The number of reads (Y-axis) was used as a proxy for abundance. Scientific exponential notation (E-notation) was used for *Rothia*, *Leptotrichia*, and *Neisseria* boxplots. “*m*e+*n*” indicates the value of “*m* ×10^*n*^”. *P*-values of pairwise comparisons are displayed above each block analyzed (T-test, Wilcox, Tukey and ANOVA tests). Final adjusted *P*-values are shown on the lower-left corner of each plot (ANOVA).

To further compare the distribution of bacterial genera/species across the resistance groups, we performed Beta-diversity analyses using the T-test, Wilcox, Tukey, and ANOVA tests (Fig 5, S2 and S3 Figs). Although some species were more abundant in specific resistance groups, multiple pairwise comparisons indicate no statistically significant correlations (Adjusted *P*-values- ANOVA) for most identified genera, with the exception of Haemophilus which was associated with the presence of the TEM and others genes (Fig 5).

However, the examination of two groups at a time revealed significant associations. The genera/species *Actinomyces odontolyticus*, *Leptotrichia* spp., and *Fusobacterium* were most strongly correlated to the “SUSC” and “Others” groups.

The extended error bar plots show significant differences between the mean proportions of selected bacterial species in these two groups (S3 Fig). *Campylobacter concisus* (*p* = 0.029), *Streptococcus intermedius* (*p* = 0.026), *Rothia dentocariosa* (*p* = 0.031), *Prevotella pallens* (*p* = 0.041) and *Actinomyces graevenitzii* (*p* = 0.026) were associated with “SUSC” samples. On the other hand, *Actinomyces odontolyticus* (*p* = 0.029) was correlated with the “Others” group.

## Discussion

PD are biofilm-associated infections that stem from complex interactions between oral microorganisms and the host immune response [37]. Differently from other bacterial diseases, PD are not caused by a single pathogen. Conversely, they are triggered by a change within the oral microbiota composition; a phenomenon referred to as dysbiosis [2, 4]. Characterizing the microbial communities associated with health and disease is the first step towards understanding the dynamics of dysbiosis, and ultimately improving PD diagnosis and treatment.

Here we conducted a cross-sectional study to investigate the composition of the oral microbiota from healthy individuals and patients with periodontal diseases, as well as the prevalence of ARGs. Furthermore, we evaluated possible risk factors for PD and ARGs carriage.

Most of the patients included in our investigation were diagnosed with PD (77.3%). This finding is consistent with the Epidemiological Survey of Oral Health conducted by the Brazilian Ministry of Health [38], which shows that in the Northeast region, 71.24% of the population presented PD. In addition, data retrieved from the World Health Organization Oral Health Bank reveals that developing nations have a higher prevalence of PD than developed ones [39].

Their higher level of social inequality may explain the elevated prevalence of PD in the aforementioned regions [13]. There is a consistent association between lower socioeconomic status (income, occupation, and educational level) and the prevalence and severity of oral diseases [12–14]. Poor oral hygiene practices can lead to the accumulation of microbial biofilm (dental plaque) on teeth and gums. If left undisturbed, plaque calcifies to form calculus or tartar, which triggers inflammatory changes in periodontal tissues [37]. Our findings support this hypothesis since the majority of patients (71.0%) had a lower *per capita* monthly income (1–2 minimum wages), as well as a lower level of education.

Overall, the patients included in this investigation were more likely to be female. This predominance might be explained by the fact that women tend to use preventive and diagnostic services more frequently than men [40]. However, PD was proportionally higher among males (87.0% vs. 75.0% females). Those findings are in contrast to previous studies that report an increased likelihood of PD in women owing to hormonal changes before menstruation, during ovulation and pregnancy [12].

In our investigation, 16.1% of subjects were pregnant. During pregnancy, women experience several hormonal shifts. Previous evidence has shown that high estrogen and progesterone levels, associated with inadequate oral hygiene, can lead to PD [41]. Most pregnant women who enrolled in this study (78.57%) presented PD, and 50% had chronic periodontitis. The inflammatory process triggered by PD increases the risk of systemic alterations, which might complicate pregnancy [42].

It is widely described in the scientific literature that the risk of periodontal diseases increases with the advancing of age, with the incidence rising steeply in adults between 30 and 40 years old [12, 13]. In our sample, most of the participants were middle-aged; however, no statistically significant differences were observed between healthy and diseased patients.

Furthermore, we assessed other risk factors that have been extensively correlated to PD, such as smoking history, self-reported race, and underlying diseases (diabetes, hypertension, cardiopathy) [12, 14]. Nevertheless, we found no significant differences between the two groups.

In dentistry, antibiotics are prescribed to treat not only odontogenic infections (usually associated with conventional mechanical therapy) but also non-odongenic diseases. Furthermore, prophylactic antibiotics are taken before several procedures to reduce the chance of postoperative local and systemic complications. The most frequently used antibiotics are amoxicillin, amoxicillin/clavulanic acid, azithromycin, clindamycin, ciprofloxacin, gentamicin metronidazole, penicillin, and tetracycline [8, 9].

Here we aimed to determine the prevalence of antibiotic resistance among oral microorganisms. We screened genes that render resistance to antibiotics regularly used in dental practice, focusing on β–lactamics (*bla*_TEM_, *bla*_SHV_, *bla*_OXA-1-like,_ *pbp*2b_,_ *bla*_CTX-M_, *mec*A), aminoglycosides (aac (6’)), macrolides (*erm*), and metronidazole (*nim*). Although carbapenemases genes (*bla*_KPC_, *bla*_IMP_, *bla*_VIM_, *bla*_NDM_, and *bla*_OXA-48-like)_ are not frequently reported in oral bacteria, we screened these ARGs owing to their relevance in medical contexts.

In our dataset, the majority of samples (72.7%) were positive for at least one of the genes screened. The gene *erm* was the most frequent variant (58.2%). As noted from previous studies, resistance to macrolides is widespread in both commensal and pathogenic oral bacteria. One possible explanation for these high proportions of resistance is the phenomena of *co-selection* [43].

The gene *erm* is often contained on conjugative transposons from the Tn*916* family (Tn*1545*, Tn*6002*, and Tn*6079*). These mobile genetic elements harbor multiple resistant determinants. They confer resistance to different antibiotics, a few of which are tetracycline and kanamycin. Therefore, the usage of these drugs may co-select for erythromycin resistance [43, 44].

Furthermore, the presence of *erm* genes in the oral cavity is of particular clinical importance. Recent evidence has shown that these genetic determinants are often associated with some oral commensals, such as the viridans group streptococci (VGS). Using conjugative transfer, VGS can disseminate erythromycin resistance to major streptococcal pathogens, a few of which are *Streptococcus pneumoniae* and *Streptococcus pyogenes* [45].

Following *erm*, the ARGs more prevalent in our sample were *bla*TEM (16.4%), *mec*A (2.7%), *pbp*2b, and *aac*(6 ') (1.8%). These findings are similar to the ones described in the systematic review conducted by Moraes and colleagues [46]. The authors have analyzed clinical studies on the detection of resistance genes in the oral cavity. Their results revealed that the *erm* is one of the most frequently ARG, along with *bla*_TEM_, *cfxA*, and *tet* genes, indicating the external validity of our dataset.

The proportion of ARGs was higher in diseased patients. However, statistical significance was not reached. Similarly, other studies have previously reported a higher prevalence of ARGs in compromised periodontal sites [47, 48] Such findings might be explained by the abundance of gram-negative species in periodontal lesions. Gram-negative bacteria generally have a high genetic horizontal transfer rate, *i*.*e*., they have an excellent facility for exchanging DNA among strains, which leads to the ubiquity of ARGs [48].

We characterize the oral microbiome from 22 subjects. The samples were primarily selected based on their resistance patterns, including resistant and susceptible profiles.16S rRNA gene sequencing analyses revealed that the oral microbial communities were mainly composed by the phylum *Firmicutes*, *Bacteroidetes*, *Actinobacteria*, *Fusobacteria*, and *Proteobacteria*, consistent with previous studies [19, 49].

The taxonomic distribution of our amplicon-based data at the species level is also in agreement with previous findings [3, 19, 49]. *Streptococcus* and *Rothia* were the most frequently reported genera both in healthy and diseased patients. *Prevotella* was enriched in PD samples.

There were no significant differences in terms of taxonomic enrichment between the two groups. However, samples retrieved from healthy patients had a more diverse microbial community, whereas diseased samples have lower taxonomic diversity. These findings differ from previous results reported in the literature [19, 37, 49]. Our microbiome analysis was based on a limited subset of our samples (10%, *n* = 22); therefore, these results must be interpreted with caution.

The present investigation has the significant advantage of using culture-independent 16S rRNA gene sequencing to evaluate taxonomic diversity. We also have used the magnetic beads DNA extraction method, which provides optimal results.

However, this study also has some limitations that should be acknowledged.

Firstly, we set up a short period of suspension of antibiotic treatment (30 days), which may have affected the susceptibility reports. Moreover, the oral microbiome analysis was based on a small number of samples, which has limited our ability to make inferences and draw conclusions.

The majority of individuals in our dataset were female, middle-aged, and without underlying diseases and smoking history. This high level of similarities reflects one of the drawbacks of using data retrieved from cross-sectional investigations. Longitudinal designs are more appropriate to study the epidemiological aspects of PD and assess the diversity of healthy and diseased microbiomes.

## Conclusions

In summary, this study revealed that the proportion of ARGs was similar between healthy and diseased patients. The *erm*, *bla*TEM, *mecA*, and *pbp2b* were the predominant resistance determinants, indicating their spread between oral microorganisms in our setting, mainly among Haemophilus species.

The 16S rRNA gene sequencing analysis revealed differences in the taxonomic composition of healthy and diseased oral microbiomes. Healthy patients had a more diverse microbial community, which is in contrast to previous studies. However, it is important to highlight that our microbiome analysis was based on a small number of samples. To confirm our observations, further studies, with longitudinal designs and large sample sizes samples, are needed.

## Supporting information

S1 Fig Distribution of bacterial phyla in health and diseased patients. Box plots illustrate the abundance of bacterial phyla across the three groups of patients. Overall, 22 samples were analyzed being 4 (18.2%) retrieved from healthy patients, 12 (54.5%) from those with gingivitis, and 6 (27.3%) from those with chronic periodontitis. Boxes represent the interquartile range (IQR) between the first and third quartiles (25th and 75th percentiles, respectively). The vertical line inside each box defines the median. Whiskers represent the upper and lower adjacent values. The number of reads (Y-axis) was used as a proxy for abundance. *P*-values of pairwise comparisons are displayed above each block analyzed (T-test, Wilcox, Tukey and ANOVA tests). Final adjusted *P*-values are shown on the lower-left corner of each plot (ANOVA).
(TIF)

S2 Fig Bacterial genera distribution stratified by resistance groups. Box plots illustrate the abundance of selected genus across resistance groups. Overall, 22 samples were analyzed being 4 (18.2%) positive for the *erm* gene (EMR group), 6 (27.3%) for the *bla*_TEM_ gene (TEM group), 2 (9.1%) for *mec*A and *pbp*2 genes (Others group), and 10 (45.5%) negative for all searched genes (SUSC group). The boxes represent the interquartile range (IQR) between the first and third quartiles (25th and 75th percentiles, respectively). The vertical line inside each box defines the median. Whiskers represent the upper and lower adjacent values. Gray circles are outliers. The number of reads (Y-axis) was used as a proxy for abundance. Scientific exponential notation (E-notation) was used for *Rothia*, *Leptotrichia*, and *Neisseria* boxplots. “*m*e+*n*” indicates the value of “*m* ×10^*n*^”. *P*-values of pairwise comparisons are displayed above each block analyzed. Final adjusted *P*-values are shown on the lower-left corner of each plot (ANOVA).
(TIF)

S3 Fig Variability of bacterial species between “SUSC” and “Others” resistance groups. The extended error bar plot identifies significant differences between mean proportions of selected bacterial species in SUSC (blue) and Others (orange) resistance groups. The species evaluated were *Actinomyces odontolyticus*, *Actinomyces graevenitzii*, *Prevotella pallens*, *Rothia dentocariosa*, *Streptococcus intermedius*, *Campylobacter concisus*. “SUSC” designates the group of samples negative for all searched genes (*n* = 10), whereas “Others” refers to samples that were positive for *mec*A and *pbp*2 genes (*n* = 2). Significance was calculated using the *T-test*. Corrected *P*-values are shown at right.
(TIF)

S1 Table Antibiotic resistance genes profile, stratified by periodontal health status (*n* = 110). (DOCX)

## Abbreviations

*aac(6’)*: aminoglycoside-6’-N-acetyltransferase
ARGs: Antibiotic Resistance Genes
*bla*: β-lactamase
C: Degree Celsius
CDC: Centers for Disease Control and Prevention
CI: Confidence Intervals
CT: Threshold Cycling Value
CTX-M: Active on cefotaxime, first isolated at Munich
*erm*: Erythromycin ribosomal methylase
ESβL: Extended Spectrum β-lactamase
Fiocruz: Fundação Oswaldo Cruz
IMP: Active on Imipenem
KPC: Klebsiella pneumoniae carbapenemase
*mec*A: Methicillin resistance gene; mg: Milligram
mL: Milliliter; mM: Millimolar
*nim*: Nitroimidazole resistance protein
OR: Odds Ratio
OTU: Operational Taxonomic Unit
OXA-1: Active on oxacillin–variant 1
OXA-48: Active on oxacillin–variant 48
*pbp2b*: Penicillin Binding Protein 2b
PCR: polymerase chain reaction
pH: Hydrogen potential
PSR: Simplified Periodontal Register
RDP: Ribossomal Database Project
SHV: Sulfhydryl variable
SUSC: Samples of individuals negative for any resistance genes
TEM: Temoneira
UFBA: Universidade Federal da Bahia
VIM: Verona Integron-encoded Metallo- β–lactamase
NDM: New Delhi Metallo- β-lactamase
WHO: World Health Organization
