# Supplementary figures and images for: Bacterial diversity and prevalence of antibiotic resistance genes in the oral microbiome
Source: PLoS One. 2020 Sep 29;15(9):e0239664. doi: 10.1371/journal.pone.0239664 (PMC7523989; doi:10.1371/journal.pone.0239664)

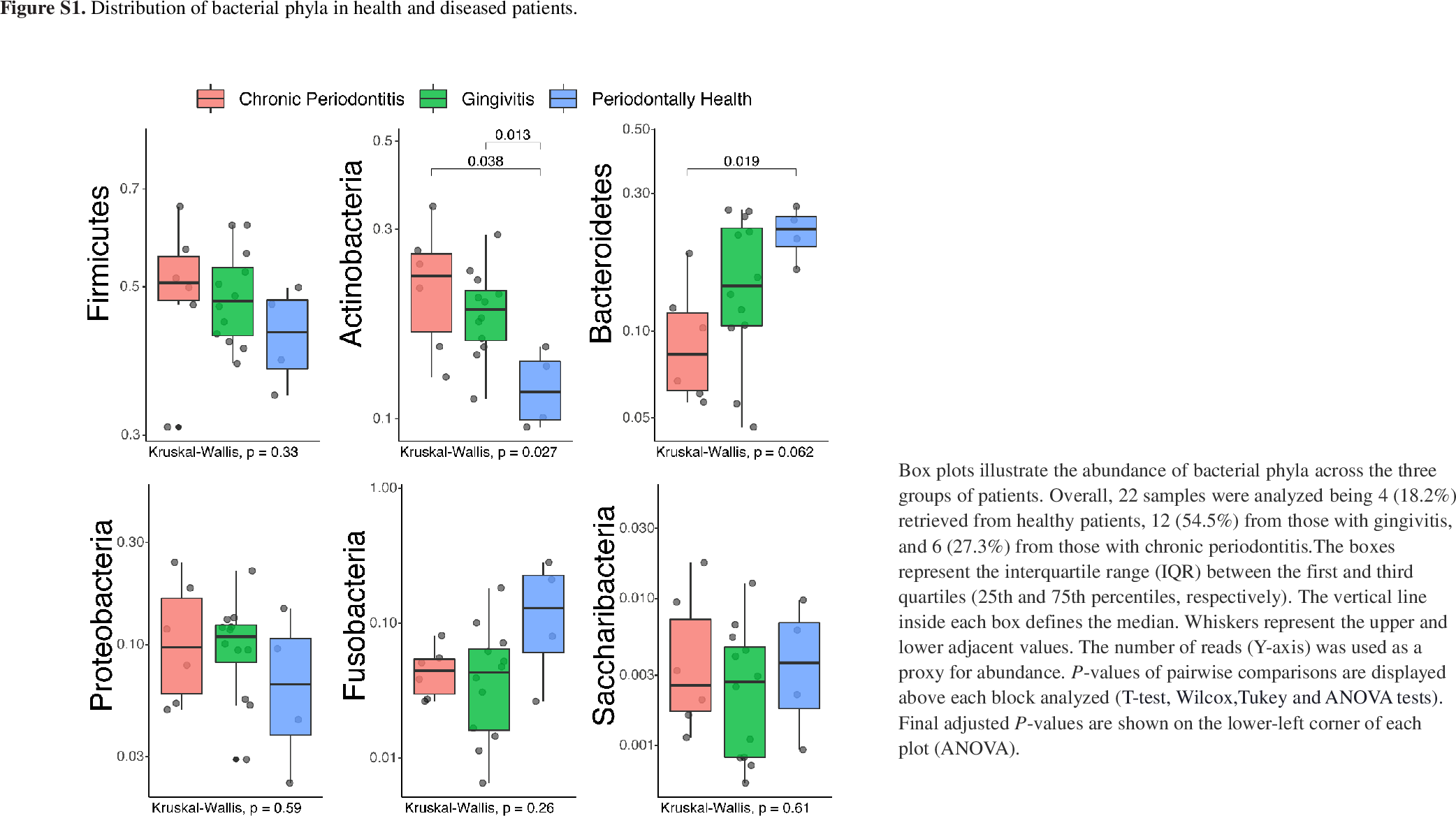

Supplement: S1 Fig — Box plots illustrate the abundance of bacterial phyla across the three groups of patients. Overall, 22 samples were analyzed being 4 (18.2%) retrieved from healthy patients, 12 (54.5%) from those with gingivitis, and 6 (27.3%) from those with chronic periodontitis. Boxes represent the interquartile range (IQR) between the first and third quartiles (25th and 75th percentiles, respectively). The vertical line inside each box defines the median. Whiskers represent the upper and lower adjacent values. The number of reads (Y-axis) was used as a proxy for abundance. P-values of pairwise comparisons are displayed above each block analyzed (T-test, Wilcox, Tukey and ANOVA tests). Final adjusted P-values are shown on the lower-left corner of each plot (ANOVA). (TIF) [file pone.0239664.s001.tif]

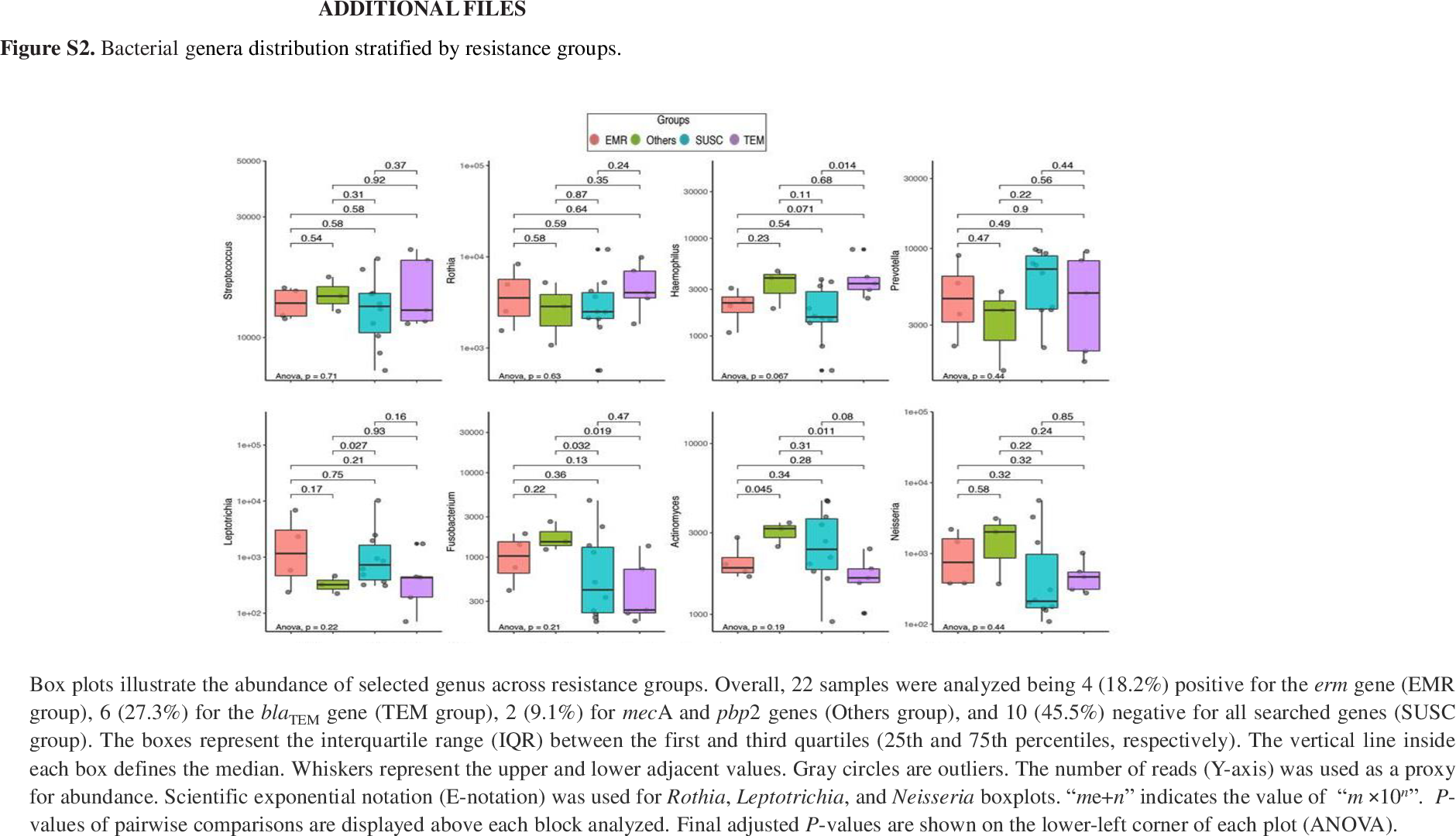

Supplement: S2 Fig — Box plots illustrate the abundance of selected genus across resistance groups. Overall, 22 samples were analyzed being 4 (18.2%) positive for the erm gene (EMR group), 6 (27.3%) for the blaTEM gene (TEM group), 2 (9.1%) for mecA and pbp2 genes (Others group), and 10 (45.5%) negative for all searched genes (SUSC group). The boxes represent the interquartile range (IQR) between the first and third quartiles (25th and 75th percentiles, respectively). The vertical line inside each box defines the median. Whiskers represent the upper and lower adjacent values. Gray circles are outliers. The number of reads (Y-axis) was used as a proxy for abundance. Scientific exponential notation (E-notation) was used for Rothia, Leptotrichia, and Neisseria boxplots. “me+n” indicates the value of “m ×10n”. P-values of pairwise comparisons are displayed above each block analyzed. Final adjusted P-values are shown on the lower-left corner of each plot (ANOVA). (TIF) [file pone.0239664.s002.tif]

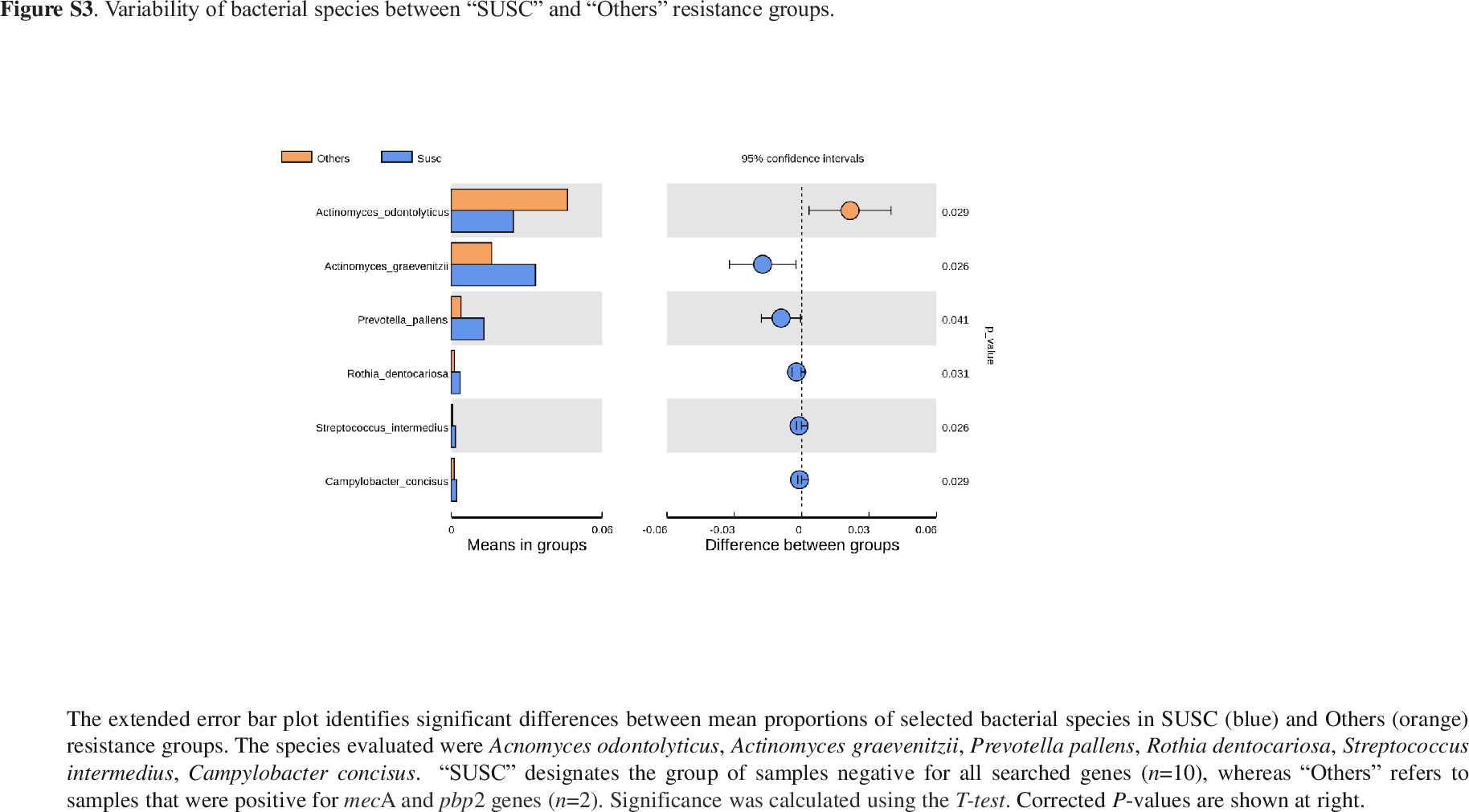

Supplement: S3 Fig — The extended error bar plot identifies significant differences between mean proportions of selected bacterial species in SUSC (blue) and Others (orange) resistance groups. The species evaluated were Actinomyces odontolyticus, Actinomyces graevenitzii, Prevotella pallens, Rothia dentocariosa, Streptococcus intermedius, Campylobacter concisus. “SUSC” designates the group of samples negative for all searched genes (n = 10), whereas “Others” refers to samples that were positive for mecA and pbp2 genes (n = 2). Significance was calculated using the T-test. Corrected P-values are shown at right. (TIF) [file pone.0239664.s003.tif]
