# Supplementary material for: Bacterial diversity and prevalence of antibiotic resistance genes in the oral microbiome
Source: PLoS One. 2020 Sep 29;15(9):e0239664. doi: 10.1371/journal.pone.0239664 (PMC7523989; doi:10.1371/journal.pone.0239664)
Supplement: S1 Table — (DOCX) [file pone.0239664.s004.docx]

**S1 Table** - Antibiotic resistance genes profile, stratified by periodontal health status (*n*=110).

| **Sample** | **PSR** | **PCR ARGs (+)** | **ARGs profiles for each sample (gray)** | | | | |
| --- | --- | --- | --- | --- | --- | --- | --- |
|  |  |  | **Quinolones** | **ESBL** | **Oxacilinases** | | **Macrolides** |
|  |  |  | **aac(6’)** | **TEM** | ***mec*A** | ***pbp*2b** | ***erm*B** |
| 3 | 4 | Yes |  |  |  |  |  |
| 4 | 2 | Yes |  |  |  |  |  |
| 6 | 2 | Yes |  |  |  |  |  |
| 7 | 3 | Yes |  |  |  |  |  |
| 9 | 2 | Yes |  |  |  |  |  |
| 17 | 1 | Yes |  |  |  |  |  |
| 18 | 0 | Yes |  |  |  |  |  |
| 19 | 2 | Yes |  |  |  |  |  |
| 20 | 2 | Yes |  |  |  |  |  |
| 21 | 0 | Yes |  |  |  |  |  |
| 22 | 0 | Yes |  |  |  |  |  |
| 23 | 2 | Yes |  |  |  |  |  |
| 24 | 4 | Yes |  |  |  |  |  |
| 26 | 1 | Yes |  |  |  |  |  |
| 28 | 2 | Yes |  |  |  |  |  |
| 29 | 0 | Yes |  |  |  |  |  |
| 30 | 2 | Yes |  |  |  |  |  |
| 31 | 2 | Yes |  |  |  |  |  |
| 32 | 1 | Yes |  |  |  |  |  |
| 33 | 0 | Yes |  |  |  |  |  |
| 34 | 1 | Yes |  |  |  |  |  |
| 35 | 0 | Yes |  |  |  |  |  |
| 36 | 2 | Yes |  |  |  |  |  |
| 37 | 2 | Yes |  |  |  |  |  |
| 38 | 4 | Yes |  |  |  |  |  |
| 39 | 1 | Yes |  |  |  |  |  |
| 41 | 1 | Yes |  |  |  |  |  |
| 42 | 2 | Yes |  |  |  |  |  |
| 43 | 3 | Yes |  |  |  |  |  |
| 44 | 2 | Yes |  |  |  |  |  |
| 45 | 2 | Yes |  |  |  |  |  |
| 46 | 4 | Yes |  |  |  |  |  |
| 47 | 2 | Yes |  |  |  |  |  |
| 48 | 2 | Yes |  |  |  |  |  |
| 50 | 2 | Yes |  |  |  |  |  |
| 51 | 3 | Yes |  |  |  |  |  |
| 52 | 3 | Yes |  |  |  |  |  |
| 54 | 1 | Yes |  |  |  |  |  |
| 55 | 1 | Yes |  |  |  |  |  |
| 58 | 0 | Yes |  |  |  |  |  |
| 60 | 2 | Yes |  |  |  |  |  |
| 61 | 3 | Yes |  |  |  |  |  |
| 62 | 2 | Yes |  |  |  |  |  |
| 63 | 0 | Yes |  |  |  |  |  |
| 64 | 3 | Yes |  |  |  |  |  |
| 65 | 3 | Yes |  |  |  |  |  |
| 67 | 1 | Yes |  |  |  |  |  |
| 70 | 0 | Yes |  |  |  |  |  |
| 73 | 3 | Yes |  |  |  |  |  |
| 74 | 1 | Yes |  |  |  |  |  |
| 75 | 2 | Yes |  |  |  |  |  |
| 76 | 0 | Yes |  |  |  |  |  |
| 77 | 3 | Yes |  |  |  |  |  |
| 79 | 2 | Yes |  |  |  |  |  |
| 80 | 2 | Yes |  |  |  |  |  |
| 81 | 4 | Yes |  |  |  |  |  |
| 82 | 0 | Yes |  |  |  |  |  |
| 83 | 2 | Yes |  |  |  |  |  |
| 84 | 0 | Yes |  |  |  |  |  |
| 85 | 2 | Yes |  |  |  |  |  |
| 86 | 2 | Yes |  |  |  |  |  |
| 87 | 2 | Yes |  |  |  |  |  |
| 89 | 2 | Yes |  |  |  |  |  |
| 90 | 0 | Yes |  |  |  |  |  |
| 91 | 0 | Yes |  |  |  |  |  |
| 92 | 3 | Yes |  |  |  |  |  |
| 93 | 4 | Yes |  |  |  |  |  |
| 94 | 0 | Yes |  |  |  |  |  |
| 95 | 1 | Yes |  |  |  |  |  |
| 97 | 0 | Yes |  |  |  |  |  |
| 98 | 3 | Yes |  |  |  |  |  |
| 99 | 2 | Yes |  |  |  |  |  |
| 101 | 0 | Yes |  |  |  |  |  |
| 102 | 0 | Yes |  |  |  |  |  |
| 103 | 2 | Yes |  |  |  |  |  |
| 104 | 2 | Yes |  |  |  |  |  |
| 105 | 1 | Yes |  |  |  |  |  |
| 107 | 2 | Yes |  |  |  |  |  |
| 108 | 1 | Yes |  |  |  |  |  |
| 109 | 2 | Yes |  |  |  |  |  |

# All individuals (*n* = 110) samples were negative for the following ARGs investigated by PCR: *bla*_SHV_, *bla*_OXA-1-like_, *bla_CTX-M_*, *bla*_KPC_, *bla*_IMP_, *bla*_VIM_, *bla*_NDM_, *bla*_OXA-48-like_, and *nim*

# ARG: Antimicrobial resistance gene; PSR: Simplified Periodontal Register
